# How can we use stem cell-derived cardiomyocytes to understand the involvement of energetic metabolism in alterations of cardiac function?

**DOI:** 10.3389/fmmed.2023.1222986

**Published:** 2023-09-01

**Authors:** Sabine Rebs, Katrin Streckfuss-Bömeke

**Affiliations:** ^1^ Institute of Pharmacology and Toxicology, University of Würzburg, Würzburg, Germany; ^2^ Clinic for Cardiology and Pneumology, University Medicine Göttingen and DZHK (German Centre for Cardiovascular Research), Göttingen, Germany; ^3^ Department of Translational Research, Comprehensive Heart Failure Center (CHFC), University Clinic Würzburg, Würzburg, Germany

**Keywords:** mitochondrial cardiomyopathy, iPSC-cardiomyocytes, maturation strategies, Barth syndrome, Friedreich’s ataxia, lysosomal storage disorders

## Abstract

Mutations in the mitochondrial-DNA or mitochondria related nuclear-encoded-DNA lead to various multisystemic disorders collectively termed mitochondrial diseases. One in three cases of mitochondrial disease affects the heart muscle, which is called mitochondrial cardiomyopathy (MCM) and is associated with hypertrophic, dilated, and noncompact cardiomyopathy. The heart is an organ with high energy demand, and mitochondria occupy 30%–40% of its cardiomyocyte-cell volume. Mitochondrial dysfunction leads to energy depletion and has detrimental effects on cardiac performance. However, disease development and progression in the context of mitochondrial and nuclear DNA mutations, remains incompletely understood. The system of induced pluripotent stem cell (iPSC)-derived cardiomyocytes (CM) is an excellent platform to study MCM since the unique genetic identity to their donors enables a robust recapitulation of the predicted phenotypes in a dish on a patient-specific level. Here, we focus on recent insights into MCM studied by patient-specific iPSC-CM and further discuss research gaps and advances in metabolic maturation of iPSC-CM, which is crucial for the study of mitochondrial dysfunction and to develop novel therapeutic strategies.

## 1 Introduction

Mitochondria are double-membraned organelles with various crucial functions in the eukaryotic cell. Most notably, they generate and supply cellular energy via oxidative phosphorylation. Mitochondrial dysfunction can arise as a primary cause due to a genetic mutation or as a secondary consequence ofan existing disease or other influences (environmental, toxins, etc.) (Pavez-Giani and Cyganek, 2021). The mitochondrial genome (mtDNA) codes for 37 genes important for oxidative phosphorylation. The outstanding proteins and enzymes needed for mitochondrial respiration and homeostasis are encoded by the nuclear DNA. In contrast to the linear nuclear DNA, which has a defined copy in every nucleus of a cell, the circular mtDNA can be present as various copy numbers within mitochondria (Wallace and Chalkia, 2013). Since the heart is an organ with high energy demand and hence high mitochondria content, mitochondrial aberrations affect the heart severely in its function. One in every three multisystemic mitochondrial syndromes shows an effect on the heart (Mazzaccara et al., 2021). Mitochondrial cardiomyopathy (MCM), a collective term that describes a disease of the heart muscle that is primarily driven by a defect in mitochondrial function, increasingly becoming the focus of research studies. Prominent examples of genetically caused MCM include the Barth syndrome, caused by a mutation in the *TAFAZZIN* (*TAZ*) gene (Dudek and Maack, 2017), Friedreich’s ataxia, caused by a mutation in the *FRATAXIN*(*FXN*) gene (Punga and Buhler, 2010) and propionic acidemia, caused by a mutation in the enzyme propionyl-CoA carboxylase (Wongkittichote et al., 2017).

Human induced pluripotent stem cells (iPSC) provide a unique platform for investigating various cardiac diseases *in vitro* (Itzhaki et al., 2011; Streckfuss-Bomeke et al., 2017). Somatic donor or patient cells can be reprogrammed into iPSC (Takahashi et al., 2007). Due to their pluripotent nature, iPSCs can be differentiated into various cardiovascular cell types, including ventricular or atrial iPSC-cardiomyocytes (iPSC-CM), which are one of the important cell types for the analysis of MCM (Burridge et al., 2014; Cyganek et al., 2018). In addition, other cardiac-relevant non-CM can be produced such as endothelial cells, cardiac fibroblasts or macrophages (Lyadova et al., 2021; Thomas et al., 2021). This stem cell platform allows for broad applications in clinical drug toxicity testing, regenerative medicine (Tiburcy et al., 2017), and the recapitulation of the predicted phenotypes in a dish on a patient-specific level (Prondzynski et al., 2022).

In the present review, we explore and discuss the advantages and limitations of iPSC-CM to model and study MCM and summarize the current knowledge derived from iPSC studies.

## 2 Producing cardiomyoctes from induced pluripotent stem cells (iPSC)

The general approach to produce ventricular-iPSC-CM is to sequentially manipulate the canonical Wnt pathway. Like the physiological cardiogenesis, an initial activation of Wnt drives the iPSC into a mesodermal commitment and the subsequent inhibition of Wnt guides the cells into the cardiac progenitor fate (Klaus et al., 2007). While all differentiation protocols follow this approach, different strategies for substances (growth factors versus small molecules), incubation times and concentrations, and 2D versus 3D exist. In general, the differentiation approaches can be divided into two: 1) The growth factor approach, using physiological growth factors like Activin-A, BMP4 and bFGF (Kattman et al., 2011). 2) The small molecule approach using artificial small molecules to induce (CHIR99021) and inhibit the canonical Wnt pathway (IWP) (Lian et al., 2013). Typically, Wnt is activated for 1–3 days, followed by a 1–3 days Wnt inhibition phase. In general, both strategies yield functional beating iPSC-CM of comparable quality, which is extensively reviewed elsewhere (Lyra-Leite et al., 2022). Recent advances in this field comprise approaches to mix growth factors with small molecules or refine the substance cocktail to get more specific cardiac progenitor cells (Zawada et al., 2023). Here, retinoic acid plays a key role to produce specific cardiac progenitor cells that give rise to different cardiac cells like left ventricular cells, right ventricular cells, atrial cells or cells of the outflow tract (Cyganek et al., 2018; Zawada et al., 2023). Although differentiation protocols are becoming more optimized, iPSC-CM still need to be purified. This is achieved either by a metabolic selection step, as CM can survive with lactate as a carbon source while other cell types are starved (Tohyama et al., 2013), or the cells are fluorescently or magnetically sorted using a cardiac specific marker (Dubois et al., 2011). The strengths and limitations of these in vitro-derived iPSC-CM are discussed in the next two chapters Section 2.1 and Section 2.2.

### 2.1 Strengths of iPSC-CM technology

The iPSC-platform holds many advantages to study mitochondrial structure and function in a disease context and circumvents some existing limitations of current traditional models. First advantage of the iPSC technology is the unlimited source of human cardiac cell types, which can be maintained in cell culture over months for broad applications in clinical drug efficacy and toxicity testing, or regenerative medicine (Tiburcy et al., 2017). Second, patient-specific iPSC can be obtained from any patient and allow the investigation of a mitochondrial phenotype even if the underlying genetic cause is still unclear. Therefore, the genetic identity to their donors is maintained encompassing both nuclear and mtDNA background and enables a robust recapitulation of the predicted phenotypes in a dish on a patient-specific level (Prondzynski et al., 2022). Advances in gene editing methods such as CRISPR/Cas9 and TALENs allow to purposefully manipulate iPSC to generate isogenic rescue iPSC from a patient line or introduce a mutation into a control line (Gahwiler et al., 2021). This is a crucial advantage if, e.g., a mutation is known but no willing donor is available. Directed differentiation protocols allow to generate a multitude of different cell types. In cardiac research, ventricular, atrial, and nodal cardiomyocytes (CM) as well as other cardiac relevant non-myocytes (endothelial cells, cardiac fibroblasts, smooth muscle cells) can be produced and provide a valid human cell system (Cyganek et al., 2018; Giacomelli et al., 2020; Lyra-Leite et al., 2022). Because this is a human cell platform, interspecies differences derived from animal models can be circumvented. Animal models do not fully recapitulate the human cardiac physiology and especially in the context of mtDNA-mutation-based mitochondrial cardiomyopathy (MCM) available mouse models are sparse (Kauppila et al., 2016). A particular advantage of the iPSC model is seen in the study of mitochondrial diseases arising from mutations in mtDNA. In the case of a heterozygous mtDNA mutation one cell possess copy numbers of both wildtype and mutated mtDNA in various ratios (heteroplasmy). This heteroplasmy is one of the main reasons for different disease outcomes and manifestations. It has been shown that reprogramming of somatic cells into iPSC randomly produces iPSC lines with different copy ratios of healthy to mutated mtDNA content (Klein Gunnewiek et al., 2020). This allows the direct influence of heteroplasmy on disease manifestation to be studied and correlated.

### 2.2 Limitations of iPSC-CM technology in the analysis of metabolic dysfunction

Although considerable progress in CM derivation has been made, *in vitro* derived iPSC-CM resemble an immature, neonatal status of cardiac cells and show differences in cellular and functional parameters compared to isolated adult primary CM. This problem is well-known and maturation strategies for iPSC-CM have been addressed on multiple levels, with the most common approach being long-term culture, the addition of molecular modulators or substrates, electrical or mechanical stimulation, more dimensional heart organoids or a combination of different approaches (Liaw and Zimmermann, 2016; Tiburcy et al., 2017; Ronaldson-Bouchard et al., 2018; Ahmed et al., 2020; Emanuelli et al., 2022). The maturation parameters used were adopted from the knowledge of human cardiogenesis. This includes cell size and shape, expression of cardiac specific markers such as α-actinin or cardiac troponin, electrophysiological properties and resting membrane potential, sarcomeric organization and length and force generation (Thomas et al., 2022). Many studies have focused on these parameters to verify whether a particular maturation protocol is effective. However, metabolic maturation was often neglected in previous studies and is now increasingly becoming the focus of investigation, especially in the context of mitochondrial diseases. The parameters of mitochondrial maturation are much more difficult to define clearly because of the lack of detailed knowledge of human adult CM. For example, it is widely accepted that a mature cardiomyocyte has a rod-like shape, a very regular sarcomeric pattern, a cell type specific action potential, and a resting membrane potential of −90 mV (Yang et al., 2014; Denning et al., 2016). In contrast, the mitochondrial membrane potential ΔΨm is difficult to determine in absolute terms, is often derived from animal models, and differs when cells or isolated mitochondria are measured (Ramzan et al., 2010). The value of ΔΨm is considered to be ∼150 mV, although comprehensive data on ΔΨm during human heart development are lacking (Davidson et al., 2007). In most cases, the ΔΨm is measured in relative values compared to a control condition, and an increase in ΔΨm is usually interpreted as good/healthy/mature.

It is defined that the adult heart relies mainly on fatty acid oxidation (FAO) to produce ATP. However, it is difficult to define what ratio of glycolysis to FAO is an appropriate value for a fully mature and adult CM. Moreover, human data showing the complete transition from a postnatal to a fully adult metabolic profile are lacking. Nevertheless, it is now generally accepted that a decrease in glycolysis and an increase in FAO with a concomitant increase in ATP production is a reliable indication of metabolic and thus mitochondrial maturation. Other parameters used to study mitochondrial development include mitochondrial size, shape, content, cristae formation, and network architecture. In an adult CM, the mitochondrial network is aligned with the sarcomere for efficient ATP supply and represents a rather rigid and undynamic structure in the adult heart (Li et al., 2020). A CM dedicates ∼30% of its cell volume to mitochondria, with ∼7000 mitochondria present in a ventricular CM (Dedkova and Blatter, 2012). In addition to the main function of ATP generation, mitochondria are also important players in ROS production and have a physiological calcium homeostasis. In a previous study, increased ROS levels along with an increase in ΔΨm were interpreted as indicative of increased mitochondrial content and function (Kim et al., 2022). Although CM have numerous mitochondria, they have relatively low ROS levels in a healthy physiological state (Garbern and Lee, 2021). This underscores the difficulty of interpreting these parameters in a developmental context.

### 2.3 Strategies for mitochondrial maturation of iPSC-CM

In recent years, attention to metabolic and mitochondrial maturation for iPSC-CM approaches has increased. This is due to the fact that MCM have become a major focus in cardiovascular medicine. In addition, it has been proposed that mitochondrial maturation also drives overall cardiac maturation, as incubation with a fatty acid (FA)-containing “maturation” media could also improve sarcomere structure and force development (Horikoshi et al., 2019; Yang et al., 2019). Some studies have emphasized the importance of the maturation status of iPSC-CM, because cardiac functional deficits are associated to a certain maturation status of these cells. Feyen and colleagues elegantly showed that the well-known contractility defect of RBM20 mutation-based DCM was only clearly observed when RBM20-iPSC-CM were treated with FA-based maturation medium (Feyen et al., 2020). In contrast, Cui and colleagues described the effects of Doxorubicin (Dox) on 30 days vs. 60 days old iPSC-CM and demonstrated that immature CM of 30 days are more sensitive to DOX as a result of a higher concentration of topoisomerase IIα, which leads to more DNA damage compared to 60 days old iPSC-CM (Cui et al., 2019).

Reports focusing on the enhancement and study of mitochondrial maturation are summarized in Supplementary Table S1A. In 2013, a detailed study showed that prolonged culture time significantly promoted the structural and sarcomeric maturation of CM (Kamakura et al., 2013). Further studies have recently reported that prolonged culture time also matures the metabolic profile in iPSC-CM. A culture time up to 100 days increased the ΔΨ_m_ and the mitochondrial content (Dai et al., 2017). Emanuelli and colleagues compared in detail the metabolic profile between 6- and 12-week-old iPSC-CM and described that although glycolysis is still the main source of energy, glycolysis is coupled to OXPHOS and not to lactate production. In addition, they also observed an increase in ΔΨm and in mitochondrial network branching and length (Emanuelli et al., 2022). Another promising approach for iPSC-CM maturation was the addition of FA to the culture medium to mimic the physiological environment for the iPSC-CM. It quickly became apparent that FA alone did not trigger maturation processes and that additional stimuli were necessary (Funakoshi et al., 2021). Addition of a FA combination of palmitic acid, oleic acid and linoleic acid together with carnitine supplementation results in an overall iPSC-CM maturation with rod shaped CM with a regular sarcomere pattern and increased force development. However, the only metabolic measurement in this study describes that maximal respiratory capacity is increased (Yang et al., 2019). A much more detailed metabolic study by Funakoshi and colleagues identified the mixture of PA, PPARα agonist, dexamethasone and T3 hormone into an effective PPDT-cocktail to induce FAO. Interestingly, they demonstrated that only a transient induction with PPDT over 9 days activated mature FAO of exogenous FA, whereas continuous 2-week PPDT treatment induced FAO of endogenous FA and accumulation of lipid droplets, which is more consistent with a neonatal phenotype. This transient treatment with PPDT also resulted in larger mitochondria with more cristae structures. Moreover, this protocol can also be applied to atrial iPSC-CM (Funakoshi et al., 2021). Another study from the Dubois group reported a similar approach but concluded that a longer incubation of 4 weeks with a mixture of PA, OA and LA together with a PPARδ agonist is an applicable strategy to increase FAO, maximal and spare respiration capacity, filamentous network, mitochondrial content, mitochondrial surface area and cristae structures (Wickramasinghe et al., 2022). Other studies reported that molecular modulators of HIF1α or AMPK activity or even the plant-based tomatidine can induce metabolic maturation (Hu et al., 2018; Ye et al., 2021; Kim et al., 2022).

It remains a challenge to apply the correct maturation strategy for disease modelling with iPSC-CM and so far, no maturation protocol was able to fully mature a sarcomere-like mitochondrial network structure (Li et al., 2020). The study of mitochondrial disease necessitates a certain degree of metabolic maturation to functionally study a mitochondrial disease phenotype. To date we still lack studies that combine and directly compare multiple (metabolic) maturation protocols, e.g., electrical stimulation with fatty acid supplemented medium and long-term culture.

## 3 Insights into mitochondrial cardiomyopathies using iPSC-CM

**FIGURE 1:**
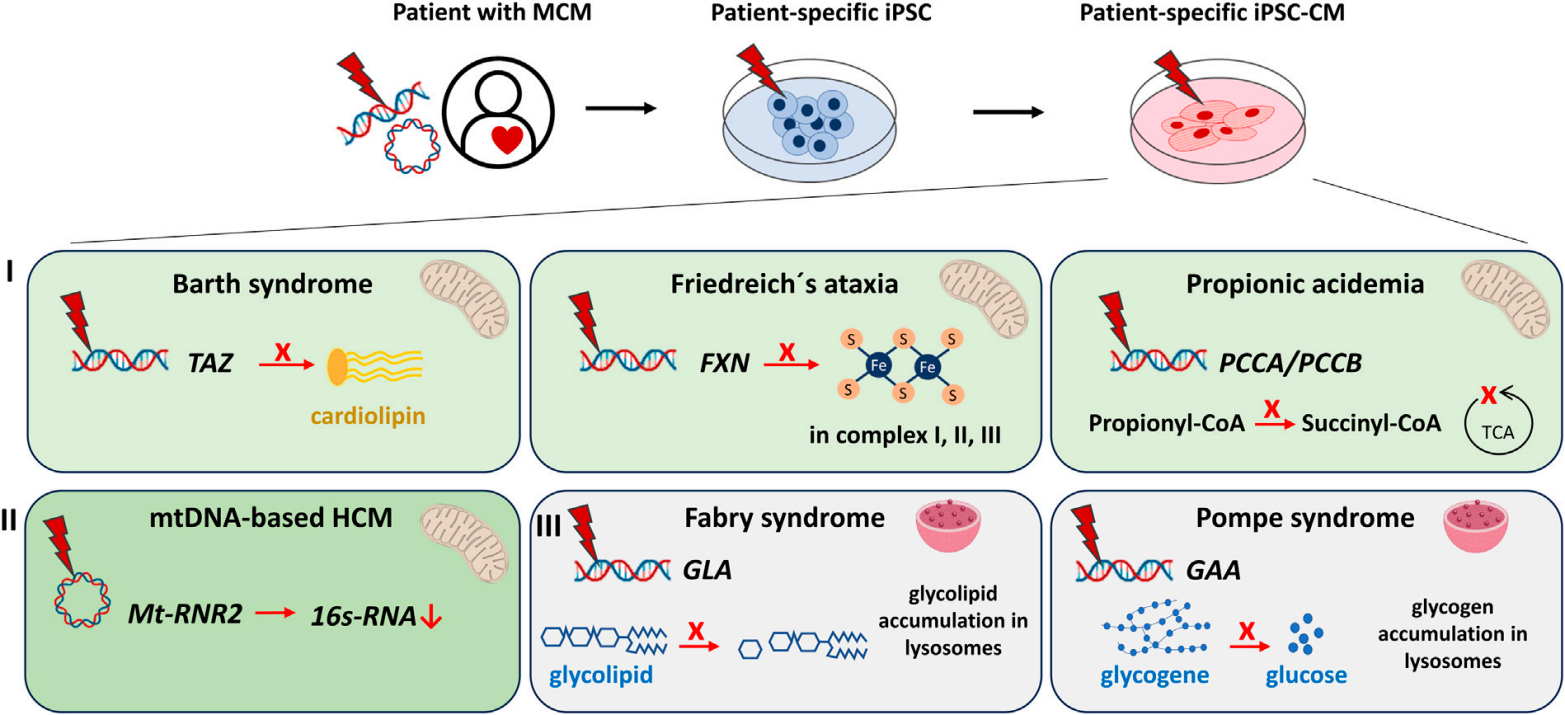
Modeling of metabolic/mitochondrial cardiomyopathies (MCM) using human iPSC‐ cardiomyocytes (iPSC‐CM) discussed in this review. MCM can be modeled *in vitro* by reprogramming somatic cells of MCM patients to iPSC and subsequent differentiation into iPSC‐CM. IPSC harbour the genetic information of the patients and the disease. Cardiac dysfunctions studied via iPSC‐CM include: I) Syndromes with cardiomyopathies caused by mutations in nuclear‐encoded mitochondria‐relevant genes: The Barth syndrome [mutation in *TAFFAZIN* (*TAZ*)] causes failed maturation of the mitochondrial cardiolipin. Friedreich’s ataxia [mutation in the *FRATAXIN* (*FXN*)] iPSC‐CM demonstrate an impaired production of functional Fe‐S clusters essential for the mitochondrial respiratory chain complexes I, II, III. Propionic acidemia [mutation in *PROPIONYL‐CoA CARBOXYLASE SUBUNIT alpha/beta* (*PCCA/PCCB*)] results in decreased production of succinyl‐CoA, an important intermediate of the mitochondrial tricarboxylic acid cycle (TCA). II) Cardiomyopathies caused by mutations of the circular mitochondrial DNA. Mutations in the *Mt‐RNR2* gene coding for mitochondrial ribosomal 16s‐RNA result in hypertrophic cardiomyopathy (HCM). III) Syndromes with cardiomyopathies that originate from lysosomal storage defects. The Fabry syndrome [mutation in *alpha‐GALACTOSIDASE A* (*GLA*)] manifests in increased glycolipid accumulation, whereas the Pompe syndrome [mutation in *alpha‐GLUCOSIDASE* (*GAA*)] concludes in over-accumulation of glycogene.

Over the past years, several studies on MCM using iPSC-CM have been conducted. As mentioned previously MCM is not an isolated cardiomyopathy, but often a cardiomyopathy that accompanies a syndromic mitochondrial disease. Prominent examples include the Barth syndrome (BTHS) and Friedreich’s ataxia syndrome (FRDA). An overall list of mtDNA and nucDNA genes contributing to mitochondrial function and corresponding mutations in a disease context have been extensively reported elsewhere (El-Hattab and Scaglia, 2016; Barca et al., 2020; Pavez-Giani and Cyganek, 2021; Caudal et al., 2022). Since these syndromes are based on nuclear or mitochondrial gene mutations with a prominent cardiac phenotype, the iPSC-CM platform provides an excellent tool to study the underlying disease drivers. Here, we focus on primary MCM that have been modelled and studied using iPSC-CM. A comprehensive summary on the studies discussed in this review is provided in Figure 1 and Supplementary Table S1B.

The **BTHS** is an inherited disease that weakens muscle tissue resulting in skeletal myopathy and cardiomyopathy. In detail, the transacylase TAZ is important in maturation of the distinctive phospholipid cardiolipin that is enriched in the mitochondrial inner membrane (IMM). Cardiolipin is an essential component within the IMM, where it binds and interacts with numerous proteins, e.g., from the respiratory chain (Dudek and Maack, 2017). In a first patient specific BTHS study, iPSC from three patients with different mutations in *TAZ* were generated. The authors demonstrated an impaired remodeling of cardiolipin, a dramatic decrease in basal oxygen consumption rate as well as the maximal respiratory capacity in BTHS-iPSC. Decreased respiration coincided with respiratory chain supercomplex remodeling leading to generation of reactive oxygen species (Dudek et al., 2013). In follow up studies, the same group confirmed the remodeling of the respiratory chain and the deficiency in succinate dehydrogenase in cardiomyocytes derived from BTHS patient’ iPSCs (Dudek et al., 2016). Furthermore, they used the same *TAZ*-deficient iPSC-CM to confirm the reduced protein expression of the pore-forming mitochondrial Ca^2+^ uniporter (MCU) subunit (Bertero et al., 2021). Wang et al. reported in 2014 that BTHS-iPSC-CM harboring two distinct mutations in *TAZ* (c.517delG and c.328T>C) replicated the immature cardiolipin processing followed by cellular energy depletion due to a dysfunctional ATP-synthase. Consequently, the BTHS-iPSC-CM exhibit characteristics of cardiomyopathy phenotype with disarrayed sarcomeric structure and weakened force generation (Wang et al., 2014). Importantly they showed that these pathologies are reversed when wt-*TAZ*-mRNA is introduced, and the pathologies are provoked if a *TAZ* mutation is introduced into a wt-iPSC line. This proved conclusively that the *TAZ* mutation is the sole and genetic background-independent driver for BTHS (Wang et al., 2014). In a following study by the same group, they linked the increased diastolic calcium and decreased calcium transient peaks to a ROS-induced hyperactivation of CAMKIIδ resulting in RYR2-Ca^2+^ leakage (Liu et al., 2021). Another study used *TAZ*-iPSC-CM harboring the same mutation (c.517delG) to uncover the influence on cardiac metabolism. In contrast to the control group, the BTHS-iPSC-CM shifted their carbon source preference to glycolysis and lactate production and reduced their uptake of FA (Fatica et al., 2019). As aberrant energy metabolism is a prominent feature in heart diseases this underscores how mitochondrial dysfunction can be an underlying driver for the development of a cardiomyopathy exemplified bythe Barth syndrome.

The Friedreich’s Ataxia (FRDA) syndrome is a neurodegenerative disorder and in the majority of cases is accompanied by hypertrophic cardiomyopathy (HCM), which is the predominant cause of death in these patients (Jensen and Bundgaard, 2012). The underlying genetic driver is a gene mutation in the *FXN* which aberrantly includes various GAA repeats within the first intron. *FXN* is a nuclear encoded gene that is crucial for mitochondrial synthesis of its Fe-S clusters in the OXPHOS. The aberrant GAA repeats do not produce a dysfunctional FXN, but rather reduce FXN drastically by inducing heterochromatin at the *FXN* locus and thus decreasing *FXN* mRNA levels (Punga and Buhler, 2010). The first FRDA-iPSC-CM disease model was published in 2013 showing ultrastructural mitochondrial defects, but no iron accumulation or sarcomeric disarray as demonstrated previously in patient’s autopsies (Hick et al., 2013). In 2014, Lee et al. presented their insights into FRDA by using iPSC-CM generated from a 35-year-old female with FRDA. They investigated physiological parameters of FRDA-iPSC-CM versus wt-iPSC-CM (healthy donor) and observed that FRDA-iPSC-CM show the characteristic decrease in *FXN* accompanied by an irregular sarcomeric pattern and mitochondria distribution. Interestingly, other molecular pathologies were only observed when the iPSC-CM were triggered with iron stress (Lee et al., 2014). Iron stress in FRDA-iPSC-CM led to iron overload, ROS increase, decrease of the iron-buffer protein ferritin, decrease in ATP production and calcium handling aberrations such as slowed kinetics, elevated diastolic calcium and diminished SR-calcium load (Lee et al., 2014). A later study using FRDA-iPSC-CM as a platform for disease modelling and drug screening reported that FRDA-iPSC-CM similarly show elevated ROS levels, intracellular iron accumulation, disorganized mitochondrial network structures, and aberrant calcium kinetics. As FRDA is suggested to be an iron overload cardiomyopathy, they used their CM system to demonstrate that the iron chelator derferiprone (DFP) is a feasible treatment option for FRDA patients as it alleviates the ROS burden and improves calcium handling kinetics (Lee et al., 2016). In regard to the genetic predisposition in FRDA a later study elegantly showed that if the GAA repeats are genetically removed from an FRDA-iPSC line, the *FXN* levels are elevated and the HCM-specific transcriptomic signature is abolished (Li et al., 2015; Li et al., 2019).

Besides oxidative phosphorylation, the most important metabolic processes in mitochondria are β-oxidation of fatty acids and catabolism of amino acids. Defects in the catabolism of amino acids causes propionic acidemia (PA) due to mutations in the enzyme for mitochondrial propionyl-CoA carboxylase (PCC). Defects in this key enzyme impede the production of the Krebs cycle intermediate Succinyl-CoA (Wongkittichote et al., 2017). The PCC consists of alpha and beta subunits, which are encoded by the *PCCA* and *PCCB* gene, respectively. Over 60 mutations for each *PCCA* or *PCCB* have been reported to date that are predominantly missense mutations or nucleotide deletions (Kraus et al., 2012). Consequently, a cardiomyopathy develops with acquired long-QT syndrome - the major cause of mortality in these patients (Grunert et al., 2013). Patient-specific PA-iPSC-CM recapitulated the biochemical hallmark of increased propionyl-carnitine levels and showed reduced basal and maximal respiratory capacity, accumulated lipid droplets, and increased ribosomal biogenesis. Interestingly, another striking observation was the change in multiple cardiac-enriched miRNAs (Alonso-Barroso et al., 2021).

Similar to these nuclear-encoded gene mutations causing MCM, one study also employed iPSC-CM to investigate HCM, which is caused by a mitochondrial gene mutation*.* ***MT-RNR2*** encodes the mitochondrial 16s-rRNA and the homoplasmic point mutation m.2336T>C is associated with a hereditary form of HCM. IPSC-CM derived from these patients could elucidate that the stability of 16s-rRNA is decreased concomitant with a reduction of mitochondrial proteins as the 16s-rRNA is crucial in formation of a functional ribosome in the mitochondria Consequently, the iPSC-CM exhibited decreased ΔΨm and ATP production and further manifested with disturbed calcium homeostasis and electrophysiological properties (Li et al., 2018).

It should be noted that iPSC-CM can also be employed to study mitochondrial dysfunctions as a secondary consequence to a primary cause. For example, in the context of diabetic cardiomyopathy (Graneli et al., 2019) or mitochondrial dysfunction due to cardiotoxic substances (Haupt et al., 2022).

Furthermore, in addition to mitochondria-related metabolic diseases, other metabolic diseases can also be investigated. These include metabolic cardiomyopathies caused by lysosomal (storage) defects, such as **Fabry disease**. A *GALACTOSIDASE-A* (*GLA*) mutation leads to accumulation of a ceramide (GL-3) due to defective degradation of shingolipids in the lysosome. This lysosomal storage disorder affects multiple organs with the heart affected in 60% of cases, leading to left ventricular hypertrophy. A Fabry disease-specific iPSC-CM model showed physiological GL-3 accumulation. Such Fabry-iPSC-CM have been used to gain mechanistic insights by analyzing the proteome and secretome or for drug screening (Kuramoto et al., 2018; Birket et al., 2019). **Pompe disease** is another lysosomal storage defect in which mutations in the *α-GLUCOSIDASE* (*GAA*) gene cause increased accumulation of glycogen. Pompe iPSC-CM have been reported to recapitulate the disease phenotypes of GAA inactivity and glycogen overaccumulation in lysosomes (Raval et al., 2015).

These examples highlight iPSC-CM as a powerful platform to study MCM and a wide array of other metabolic pathologies in a human functional cell model.

## 4 Conclusion

This article reviews the current knowledge on MCM gained by iPSC-CM studies and further discuss research gaps and advances in metabolic maturation of iPSC-CM for the development of novel therapeutic strategies Mitochondrial diseases often cause multi-organ affected syndromes with a prevalent involvement of the heart. The iPSC technology provides the unique opportunity to model and study MCM in a human patient-specific system that can be adapted to a wide-array of research questions. Previous publications have focused specifically on iPSC-CM autonomous processes, but as our knowledge on mitochondrial diseases and on heart physiology advances, the stem cell model can be adapted accordingly, e.g., by investigating the crosstalk of other relevant cardiovascular cell types and integration into 3D-engineered tissue systems. We envision that the incorporation of multicellular models will reveal previously unexplored aspects of genetic MCM. However, producing somatic cells like iPSC-CM needs validation of functionality and physiology of CM. Considering that no fully adult iPSC-CM have been generated to date, research must also advance the cardiac maturation process. Current approaches show promising results, especially regarding metabolic maturation with different strategies to foster FAO and mature the mitochondria and sarcomeres structurally. Nevertheless, iPSC-CM already represent a promising human system for modeling MCM as demonstrated here by recapitulating the disease phenotype in Barth syndrome, Friedreich’s ataxia and propionic acidemia. These studies demonstrate the potential of patient-derived iPSC-based MCM drug screening platforms for personalized medicine. We believe that the iPSC-technology will be a major step towards precision medicine based on population screening for drug responsiveness in high throughput functional pipelines and small molecule libraries targeting key cardiac pathways.

In summary, the quest for the perfect maturation strategy to achieve full adult iPSC-CM is ongoing and it would be of great value if a uniform and easy to use maturation protocol could be established and widely adopted in modelling of metabolic diseases. That would allow directly comparing studies from different labs around the world. As MCM gather an increased interest in the cardiac field, the iPSC-technology along with improved maturation approaches, as well as robust protocols to generate specialized cells and complex tissues will undoubtedly boost our understanding of MCM disease mechanisms and therapeutic options in the future.
