# Supplementary material for: How can we use stem cell-derived cardiomyocytes to understand the involvement of energetic metabolism in alterations of cardiac function?
Source: Front Mol Med. 2023 Sep 1;3:1222986. doi: 10.3389/fmmed.2023.1222986 (PMC11285589; doi:10.3389/fmmed.2023.1222986)
Supplement: Supplementary file 1 [file Table1.DOCX]

**Suppl. Table 1A:** Summary of different studies focusing on mitochondrial maturation: X (not investigated/described); FA = fatty acids, FAO= fatty acid oxidation, PA= palmitic acid, OA= Oleic acid, LA= Linoleic acid; TCA= tricarboxylic acid cycle

| **Mitochondrial:** | **Metabolic transition** | **ATP production** | **Network** | **Membrane potential (ΔΨm)** | **Size and content** | **Shape and cristae formation** |
| --- | --- | --- | --- | --- | --- | --- |
| **Postnatal to adult cardiac phenotype** | From glycolysis to FAO | 6 kg per day in adult heart mostly relying on FAO | From perinuclear network structure to global regular pattern (like sarcomere) | Often expressed in relative numbers | 30 % of cell volume, elongated and large | Compacted matrix with deep cristae |
| **Treatment with:** |  | | | | | |
| L-Carnitine + FA (PA, OA, LA)  *Yang et al., 2019* | Maximal respiration ↑ | X | X | X | X | X |
| L-Carnitine + FA (OA, LA)  *Horikoshi et al., 2019* | Basal, maximal and spare respiration ↑  FAO ↑ | ATP production ↑ | X | X | Content ↑ | X |
| PPARα agonist + PA + Dex + T3  *Funakoshi et al., 2021* | Basal and maximal respiration ↑  FAO ↑ | ATP production ↑ | X | X | Size ↑ | Cristae ↑ |
| FA (PA, OA, LA) + PPARδ agonist *Wickramasinghe et al., 2021* | Maximal and spare respiration ↑  Palmitate-FAO ↑ | X | Shift from perinuclear to global filamentous network | X | Content ↑, surface area ↑ | Cristae ↑ |
| Extended culture time 30 d vs 100 d  *Dai et al., 2017* | X | Complex I-IV activity unchanged | X | Membrane potential ↑ | Size unchanged, content↑ | X |
| Extended culture time 6 vs 12 weeks  *Emanuelli et al., 2021* | Lactate production and pentose-phosphate way unchanged; glycolysis with TCA entry ↑ | X | Network branching and branch length ↑ | Membrane potential ↑ | Content and surface area unchanged | X |
| FA (PA, OA) without glucose or HIF1α inhibition  *Hu et al., 2018* | Glycolysis ↓  Lactate ↓  Basal and spare respiration ↑ | ATP production ↑ | X | X | Content ↑ | X |
| AMPK regulation *Ye et al., 2021* | Glycolysis ↓  Oxygen consumption rate ↑, FAO ↑ | ATP production ↑ | Interconnected filamentous network | Membrane potential ↑ | Content ↑ | Cristae ↑ |
| Tomatidine  *Kim et al., 2021* | Maximum and spare respiration ↑ | ATP production ↑ | X | Membrane potential ↑ | Content ↑ with longer and larger mitochondria | Cristae ↑ |

**Suppl. Table 1B**: Summary of reports discussed in this review using the iPSC platform for mitochondrial disease modelling. DAD: Delayed afterpolarizations, APD: Action potential duration, DFP: Deferiprone, ROS: Reactive oxygen species, SR: sarcoplasmatic reticulum

| **Disease** | **Affected gene** | **Mutation** | **Consequences** | | | **Source** |
| --- | --- | --- | --- | --- | --- | --- |
|  | | | **molecular** | **physiological** | **metabolic** |  |
| **Barth Syndrome** | *TAZ* | c.590 G>T (Gly197Val);  c. 110-1 AG>AC;  c.170 G>T (Arg57Leu) | iPSC  Cardiolipin remodeling  Supercomplex remodeling  iPSC-CMs  Succinat dehydrogenase decrease;  Decrease of MCUa protein | iPSC  ROS↑  iPSC-CM  Sarcomeric disarray | iPSC and iPSC-CM  respiration↓ | iPSCs  Dudek et al., 2013  iPSC-CM Dudek et al., 2016 ;  Bertero et al., 2021 |
| **Barth Syndrome** | *TAZ* | c.517delG  c.328T>C | Mature cardiolipin↓ | Sarcomeric disarray  Myopathic engineered tissue  ROS↑ | ATP generating efficiency↓  Glycolysis↑  Oxygen consumption rate↑  Spare respiratory capacity↓ | Wang et al., 2014 |
| **Barth Syndrome** | *TAZ* | c.517delG | ROS-dependent CAMK2δ activation:  CAMK2δ-T286p↑  CAMK2δ-M281/282ox↑  PLN_T17p↑  RYR2_S2814↑ | ROS dependent:  Ca^2+^ amplitude↓  Diastolic Ca^2+^ ↑  Ca^2+^ leak↑  SR Ca^2+^ load↓ | - | Liu et al., 2021 |
| **Barth Syndrome** | *TAZ* | c.517delG | - | - | Glucose uptake↑  Lactate production↑  Palmitate uptake↓ | Fatica et al., 2019 |
| **Friedreich’s ataxia** | *FXN* | GAA repeats | - | No iron accumulation  No sarcomere disorganization | Disorganized mitochondria | Hick et al., 2013 |
| **Friedreich’s ataxia** | *FXN* | GAA repeats | FXN↓  Iron induced: Ferritin↓  TSFR1↑ | Sarcomeric disarray  Iron overload  Iron induced:  ROS↑  Ca^2+^ amplitude↓  Ca^2+^ kinetics↑  Diastolic Ca^2+^ ↑ | Disorganized mitochondria  Iron induced:  ATP production↓ | Lee et al., 2014 |
| **Friedreich’s ataxia** | *FXN* | GAA repeats | FXN ↓ | Iron induced:  ROS↑ and iron overload  =abolished with iron chelator DFP  Ca^2+^ decay kinetics improved with DFP | Iron induced:  Disorganized mitochondria network structure  Mitochondrial content↓  =improved with DFP | Lee et al., 2016 |
| **Friedreich’s ataxia** | *FXN* | GAA repeats | Production of a FA-rescue-iPSC line  =excision of GAA repeats | - | - | Li et al., 2015 |
| **Friedreich’s ataxia** | *FXN* | GAA repeats | FXN ↓  =excision of GAA repeats rescues FXN expression and the global transcriptomic profile | Lipid droplet accumulation  = rescued in edited iPSC line |  | Li et al., 2019 |
| **HCM** | *MT-RNR2* | m.2336T>C | 16s-rRNA↓  Mitochondrial proteins↓  Mitochondrial copy number↑ | Round mitochondria with fewer cristae  SR Ca^2+^ overload  L-type Ca^2+^ current↓  DAD events↑  APD↑ | Mitochondria number↑  Mitochondrial membrane potential↓  ATP production↓ | Li et al., 2018 |
| **Propionic Acidemia /Acquired Long-QT** | *PCCA* | c.1899+4_1899+7delAGTA  c.1430-?_1643+?del | PCCA protein↓  propionylcarnithine↑  protein levels↑ (HERP, GRP78, GRP75, SIG-1R, MFN2)  miRNAs↓ | Lipid droplets↑  Ribosome biogenesis↑ | Basal respiration↓  Maximal respiration↓ | Alonso-Barroso et al., 2021 |
| **Fabry syndrome** | *GLA* | c.458G>A  c.658C>T  c.7797G>C | Decreased GLA activity  5,500 proteins in proteome and secretome  LIMP2 accumulation | excitability↑  upstroke velocity↑  AP duration↓  Diastolic Ca^2+^ ↑  Ca^2+^ amplitude↑  Ca^2+^ SR load↑ | - | Birket et al., 2019 |
| **Fabry syndrome** | *GLA* | c.7797G>C | Decreased GLA activity  Globotriaosylsphingosine accumulation  ANP↑ | - | - | Kuramoto et al., 2018 |
| **Pompe syndrome** | *GAA* | c.1935C>A  c.1935C>A and c.2040+1G>T | Decreased GAA activity, increased glycogen content | Growth rate↓ | Oxygen consumption rate↓  Lactate production↓ | Huang et al., 2011 |
